# Diversity and characteristics of colonization of root-associated fungi of *Vaccinium uliginosum*

**DOI:** 10.1038/s41598-018-33634-1

**Published:** 2018-10-16

**Authors:** Hongyi Yang, Xingyu Zhao, Changli Liu, Long Bai, Min Zhao, Lili Li

**Affiliations:** 1 College of Life Sciences, Northeast Forestry University, Harbin, 150040 China; 2 Institute of Forestry Science of Heilongjiang Province, Harbin, 150081 China

## Abstract

This study investigated ericoid mycorrhizal fungi (EMF) diversity in *Vaccinium uliginosum* across its main wild distribution range in China. Fungal communities in hair roots of *V*. *uliginosum* were analyzed using Illumina MiSeq sequencing. Only 22 OTUs were assigned to putative EMF genera. *Rhizoscyphus* and *Meliniomyces* dominated EMF communities, followed by *Clavaria*, *Oidiodendron*, *Lachnum*, *Acephala*, and *Phialocephala*. There were more dark septate endophytes (DSE) reads from the Greater Khingan Mountains than from other study areas, similar to the results of the percent colonization of DSE by the magnified intersections method. Overall, high-throughput sequencing data provided a rough community-scale sketch of root-associated fungi of *V*. *uliginosum*. Two hundred and eighty slow-growing isolates were isolated from root pieces of *V*. *uliginosum*, and the isolates matched 16 fungal genera on the basis of morphological and internal transcribed spacer sequence comparison. The isolates of *Cryptosporiopsis ericae*, *Oidiodendron maius*, *Lachnum* sp., Sordariomycetes sp., and *Pleosporales* sp., formed ericoid hyphal coils via resynthesis trails. The co-existence between EMF and DSE in hair roots was observed via trypan blue staining. A putative model for the co-existence between EMF and DSE in the hair roots of *V*. *uliginosum* was proposed. We suggest that under certain environmental stresses, such as low temperature and poor available nutrients, ericoid plants may favor co-colonization by both DSE and EMF.

## Introduction

Ericaceous plants are globally distributed and are generally found in understory plant species in cold boreal forest regions^1,2^. Specific mycorrhiza, known as ericoid mycorrhiza, can be formed between ericaceous plants and several lineages of fungi. Ericoid mycorrhizae play important roles in mineral cycling and plant nutrient acquisition^1,3^, thereby increasing plant survival in nutrient-poor environments^4^.

Although only a small number of fungi have been identified as ericoid mycorrhizal fungi (EMF), the group is taxonomically diverse^2,5^. Ascomycetes, particularly those in the order Helotiales, are dominant among reported EMF^1^. *Rhizoscyphus ericae* (formerly *Hymenoscyphus ericae* and *Pezizella ericae*) and several *Oidiodendron* spp. associate widely with different Ericaceae species and are recognized as typical EMF^6,7^. Novel molecular methods have also revealed a diverse assemblage of fungi associating to hair roots colonized by EMF, including ectomycorrhiza (ECM) fungi, dark septate endophytes (DSE), and saprotrophs^8–10^. Several DSE and ECM fungi can form structures that resemble the hyphal coils of ericoid mycorrhizae in ericaceous plants^11,12^ and increasing numbers of fungal taxa forming ericoid mycorrhizae have been identified via resynthesis experiments^2,11,12^. The documented EMF include members of ascomycetes in the order Helotiales^13^, *Capronia*^14^, DSE, such as *Phialocephala fortinii*^2,12^ and basidiomycetes, such as *Clavaria*^15^ and members of Sebacinales forming the sheathed ericoid mycorrhizae^16,17^. Several culture-independent molecular methods, such as high-throughput sequencing, have also been used to assess EMF communities in plants^18,19^.

Based on the EMF ribosomal DNA internal transcribed spacer (ITS) sequences retrieved from GenBank and UNITE, Grelet *et al*.^20^ analyzed the geographical distribution of EMF, indicating that the ITS sequences data were largely skewed towards North America and Europe^20^. Insufficient sample collection from only a few regions limits our understanding of EMF diversity. Additionally, mycorrhizal status cannot be inferred from phylogenetic information alone because many putative EMF belong to functionally diverse groups and many closely related species have different lifestyle^1,2,21^. In comparison to other common mycorrhizae such as ECM and arbuscular mycorrhizae (AM), ericoid mycorrhizae remain understudied.

The flowering plant *Vaccinium uliginosum* is native to cool temperate regions. It is an important ornamental plant globally, and northeastern China is one of its most important distribution centers^22^. *V*. *uliginosum* is an important heathland plant species in the sub-arctic heath ecosystem. Understanding the diversity of EMF in the hair roots of *V*. *uliginosum* in its native habitat is thus valuable.

The aims of this study were (1) to isolate fungi from the hair roots of *V*. *uliginosum* in its native habitat of China and verify the ability of the fungi to form ericoid mycorrhizae; (2) to assess the diversity of root-associated fungi of *V*. *uliginosum* via high-throughput sequencing techniques; and (3) to observe the colonization status of DSE and EMF and assess the percent colonization of *V*. *uliginosum* hair roots for EMF or DSE.

## Results

### Microscopic examination

**Figure 1:**
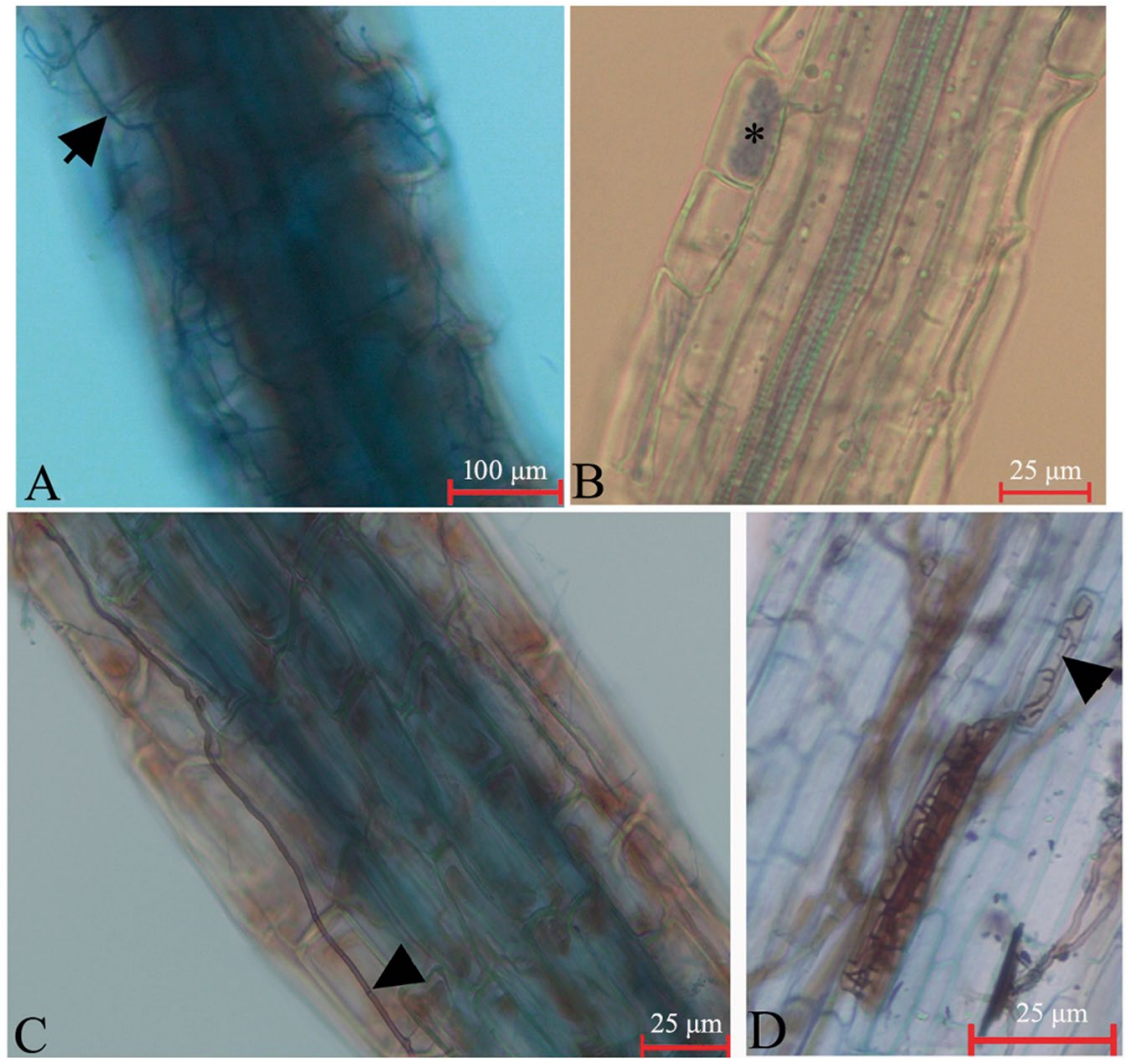
Roots of *V*. *uliginosum* colonized with fungal endophytes. (**A**) Micrograph of the root cells stained with trypan blue; a hair root of *V*. *uliginosum* coated by loose wefts of fungal hyphae, labeled with arrowhead. (**B**) A hair root colonized by fungi with coils. *Hyphal coils. (**C**) A yellowish-brown old root of *V*. *uliginosum* colonized by fungi possessing thick hyphae. The arrow indicates the fungal septate. (**D**) Microsclerotia of dark septate endophytes, labeled with an arrow.

Fine hair roots of *V*. *uliginosum* were collected from wild plants growing in the Greater Khingan Mountains, the Lesser Khingan Mountains, and the Changbai Mountains. The fungal mycelia were observed to have colonized the hairs root by light microscopy. The hair roots of *V*. *uliginosum* were coated with loose wefts of the fungal hyphae (Fig. 1A). Dense hyphal coils were observed within the cells of hair roots by trypan blue staining (Fig. 1B). This feature was identical to that previously described for EMF^23^. In addition, we frequently observed DSE, which are characterized by microsclerotia, and thick, darkly pigmented septate hyphae in the hair roots (Fig. 1C,D).

The percent colonization of hair roots by EMF or DSE was evaluated for 90 samples by the magnified intersections method. The results indicated that there were significant differences in the percent colonization of EMF among 3 study areas (Table 1). The Changbai Mountains study area was found to have the highest mean value of EMF colonization (18.5 ± 3.5%), with the cbc study site giving 21.8 ± 3.0%; while the Greater Khingan Mountains study area was found to have the lowest mean EMF colonization (12.0 ± 2.6%), with the ja study site showing as low as 11.5 ± 2.9% (Table 1).

**Table 1:** The percent colonization of hair roots by ericoid mycorrhizal fungi (EMF) and dark septate endophytes (DSE).

	EMF	DSE
Study area of the Changbai Mountains	18.6 ± 3.5a	23.6 ± 3.5c
cba study site	16.2 ± 2.3	26.0 ± 2.9
cbb study site	17.7 ± 2.6	22.0 ± 3.0
cbc study site	21.8 ± 3.0	22.8 ± 3.3
Study area of the Greater Khingan Mountains	12.0 ± 2.6c	44.3 ± 6.7a
ja study site	11.5 ± 2.9	40.5 ± 4.7
jb study site	12.4 ± 2.4	45.3 ± 6.2
jc study site	12.0 ± 2.3	47.2 ± 7.1
Study area of the Lesser Khingan Mountains	13.0 ± 2.5b	29.0 ± 5.1b
la study site	13.0 ± 2.2	30.4 ± 5.6
lb study site	14.2 ± 2.8	28.0 ± 4.3
lc study site	11.9 ± 2.0	28.4 ± 5.0

The numbers represent the mean values ± SD. Different letters indicate significantly different study areas of data (Kruskal-Wallis ANOVA + multiple comparisons, *P* < 0.05).

All test samples of *V*. *uliginosum* were colonized by DSE with a high total colonization rates of DSE. DSE colonization appeared more frequently in the yellowish-brown old hair root. There were significant differences in the total colonization rates of DSE among 3 study areas (Table 1). There were the highest colonization rates of 48.0 ± 8.4% (with the jc study site showing 47.2 ± 7.1%) for the Greater Khingan Mountains study area. Moreover, there were the lowest colonization rates of 23.6 ± 3.5% (with the cbb study site giving 22.0 ± 3.0%) for the Changbai Mountains study area.

Linear mixed model analysis indicated that there were significant differences for both EMF colonization rates and total colonization rates of DSE between different study sites, and EMF colonization rates were negatively associated with total colonization rates of DSE using study sites as a random factor (P < 0.05). Moreover, the latitude factor was also negatively associated with total colonization rates of DSE (P < 0.05). The pH factor did not have a significant effect on EMF colonization rates and total colonization rates of DSE (P > 0.05).

### Isolation and culture of root-associated fungi of *V*. *uliginosum*

We isolated 280 slow-growing fungi from the hair roots of *V*. *uliginosum*. The isolates varied in color (white, pale-gray, brown, grayish, gray, gray-black and black), but produced neither conidia nor exudate. Fluffy and flat colonies were dominant. Most isolates grew slowly; the 2-week-old colonies exhibiting irregular margins were 1–3 cm in diameter on the PDA plates. We only found vegetative mycelium in these isolates, even on the different growth media.

We selected 57 representative isolates from the different morphotype groups for the PCR analysis. The amplified ITS region and 5.8S ribosomal DNA (rDNA) region (ITS1 ± 5.8S ± ITS2) yielded products that were approximately 500 bp long. Comparisons between the obtained sequences and those present in GenBank revealed that they had more than 96% similarity to the ITS and 5.8S rDNA region of different fungal isolates (Table 2).

**Table 2:** The closest relatives of the fungal endophyte isolates based on BLAST analysis in GenBank.

Fungal isolate^&^	Best BLAST match	Similarity (%)	ERM^*^
k5, 6b, **e13** 97, 164, **k6**	*Cryptosporiopsis ericae* (JX406516)	98–100	Y
**101**	*Cryptosporiopsis ericae* (GU945547)	99	Y
70,129	*Cryptosporiopsis ericae* (KR859173)	99–100	
**103**,104, 106,114	*Cryptosporiopsis ericae* (JX406543)	99	Y
**h9**, **g5**	*Oidiodendron maius* (LC131008)	99	Y
141, **143**	*Oidiodendron* sp. (KX440134)	99	Y
7151, **151**	Uncultured *Oidiodendron* (JF519596)	99	Y
156, **140**, **119**	Uncultured *Lachnum* (FJ440910)	98–99	Y
**89**	*Lachnum* sp. (KJ817276)	99	Y
**107**,**76**	*Phialocephala fortinii* (KJ817280)	99	Y
**73**, **75**, **f5**	*Phialocephala fortinii* (JN376152)	99	Y
**t7**	*Phialocephala fortinii* (KC876262)	99	Y
**93**	*Gaeumannomyces caricis* (AJ010030)	98	N
**105**	Sordariomycetes sp. (KX909156)	99	Y
f10, **f7**, h13 h11, f15	*Phomopsis* sp. (KT264444)	99	N
e22, **f11**	*Phomopsis* sp. (KT291432)	99	N
**83**	*Chaetomium globosum* (KU936228)	99	N
**13b**	*Chaetosphaeriales* sp. (JX244060)	99	N
**t9**, **7c**	*Neonectria radicicola* (AJ875319)	99	N
**e3**	*Cylindrocarpon* sp. (AB369260)	99	N
h8, h12, **12b**	*Cylindrocarpon* sp. (GU395518)	99–100	N
**5c**, **4b**	*Botryosphaeria dothidea* (KP183181)	99	N
**k10**	*Leptosphaeria* sp. (AB752252)	99	N
**h5**, h22	*Pleosporales* sp. (KP268992)	96–98	Y
**t5b**, **f4**	*Trichoderma virens* (KU521856)	99	N
t1, **t3b**, e17	*Trichoderma virens* (JX993849)	97–99	N
**74**	*Umbelopsis isabellina* (KU847883)	97	N

*Resynthesis trials confirmed the isolates formed ericoid mycorrhizae (Y) or failed to form ericoid mycorrhizae (N). ^&^The isolates used to conduct resynthesis experiments are indicated in bold.

Based on the morphological characteristics and ITS sequence comparisons, the isolates were divided into 16 groups, matching 16 fungal genera (Table 2). The isolates included members of the class Leotiomycetes (*Cryptosporiopsis ericae*, *Oidiodendron maius*, *Lachnum* sp., and *Phialocephala fortinii*), members of the class Dothideomycetes (*Botryosphaeria dothidea*, *Leptosphaeria* sp., and *Pleosporales* sp.), members of the class Sordariomycetes (*Gaeumannomyces caricis*, *Chaetomium globosum*, *Neonectria radicicola*, Sordariomycetes sp., *Phomopsis* sp., *Chaetosphaeriales* sp., *Cylindrocarpon* sp., and *Trichoderma virens*), and a member of the order Umbelopsidales (*Umbelopsis isabellina*). The most abundant isolate was *C*. *ericae*, of which there were 13 isolates (Table 2), representing 22.8% of the total. The second most common isolate was *Phomopsis* sp., which represented 12.3% of all isolates. Two other genera, *Phialocephala* and *Oidiodendron*, represented >10% of all isolates. Among the sequenced isolates, 13 isolates exhibited high sequence similarity (98–100%) with the strains of *C*. *ericae* from GenBank, most of which were isolated from Chinese *Vaccinium* plants. Six isolates had high sequence similarity (99%) with the strains of *O*. *maius* from GenBank, suggesting that these isolates might be *O*. *maius*. Similarly, seven isolates had high sequence similarity (99%) with the strains of *Phomopsis* sp. from GenBank, indicating that they might be conspecifics of *Phomopsis*. In addition, six isolates matched (with 99% similarity) *P*. *fortinii*, which is a common species of DSE. All isolated fungi belonged to ascomycetes, with the exception of isolate 74, which had a similarity of 97% with two strains of *U*. *isabellina* (Mucoromycotina).

### Resynthesis of mycorrhizae

Nineteen of the 36 isolates formed ericoid mycorrhizal coils in the epidermal cells of hair roots four months after the inoculation of *V*. *uliginosum* seedlings under gnotobiotic conditions, and thus were considered to be ericoid mycorrhizal endophytes. Seventeen isolates did not form ericoid mycorrhizae in the resynthesis experiments (Table 2). Intracellular hyphal coils were detected in the synthesized ericoid mycorrhizae. The culture characteristics of re-isolated fungi from the synthesized ericoid mycorrhizae were observed, and the re-isolated fungi produced the same colonies as those of the original isolates.

**Figure 2:**
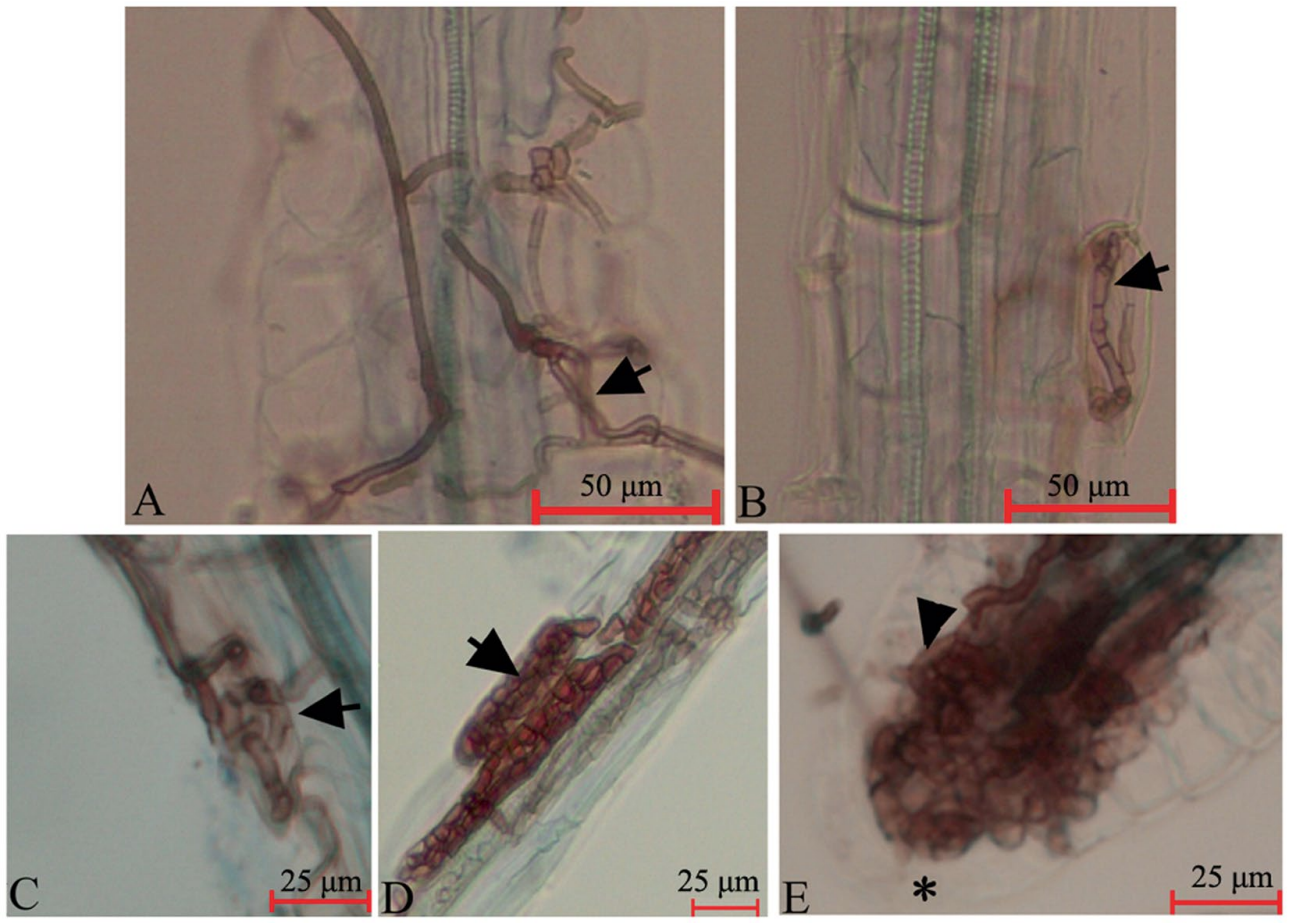
Dark septate endophytes (DSE) stained by trypan blue in *V*. *uliginosum* root tissue. (**A**) Intercellular hyphae formed hyphal nets, labeled with an arrow. (**B**) Intracellular darkly pigmented septate hyphae, labeled with an arrow. (**C**) Loosely intertwined intracellular hyphae. Arrows indicate the twined regions. (**D**) Microsclerotia of DSE, labeled with an arrow. (**E**) DSE, labeled with an arrowhead, colonized the root apex. *Undifferentiated cells.

The isolates of *P*. *fortinii* formed intercellular, thick, and darkly pigmented septate hyphae, which were long runner hyphae, typically running parallel to the root’s long axis (Fig. 2A), during he resynthesis trials. The intercellular hyphae frequently formed hyphal nets in the various tissues and sometimes branched at a 90° angle to the main hyphae (Fig. 2A). Intracellular darkly pigmented septate hyphae were observed often (Fig. 2B). The isolates of *P*. *fortinii* formed structures resembling ericoid hyphal coils (Fig. 2C). In general, the intracellular hyphae of *P*. *fortinii* were thicker and exhibited looser twining patterns. Microsclerotia were also observed in the roots of *V*. *uliginosum* (Fig. 2D). DSE colonized different tissues, such as the root apex (Fig. 2E).

Overall, the isolates of *C*. *ericae*, *O*. *maius*, *Lachnum* sp., Sordariomycetes sp., and *Pleosporales* sp. formed ericoid mycorrhizal coils, while the isolates of *P*. *fortinii* formed structures resembling ericoid hyphal coils. However, the isolates of *G*. *caricis*, *C*. *globosum*, *N*. *radicicola*, *Phomopsis* sp., *Chaetosphaeriales* sp., *Cylindrocarpon* sp., *B*. *dothidea*, *Leptosphaeria* sp., *T*. *virens*, and *U*. *isabellina* did not form hyphal coils (Table 2), and we did not find that the isolates could form other types of mycorrhizal associations in the roots of *V*. *uliginosum*.

### Observation of ericoid mycorrhizae by TEM

**Figure 3:**
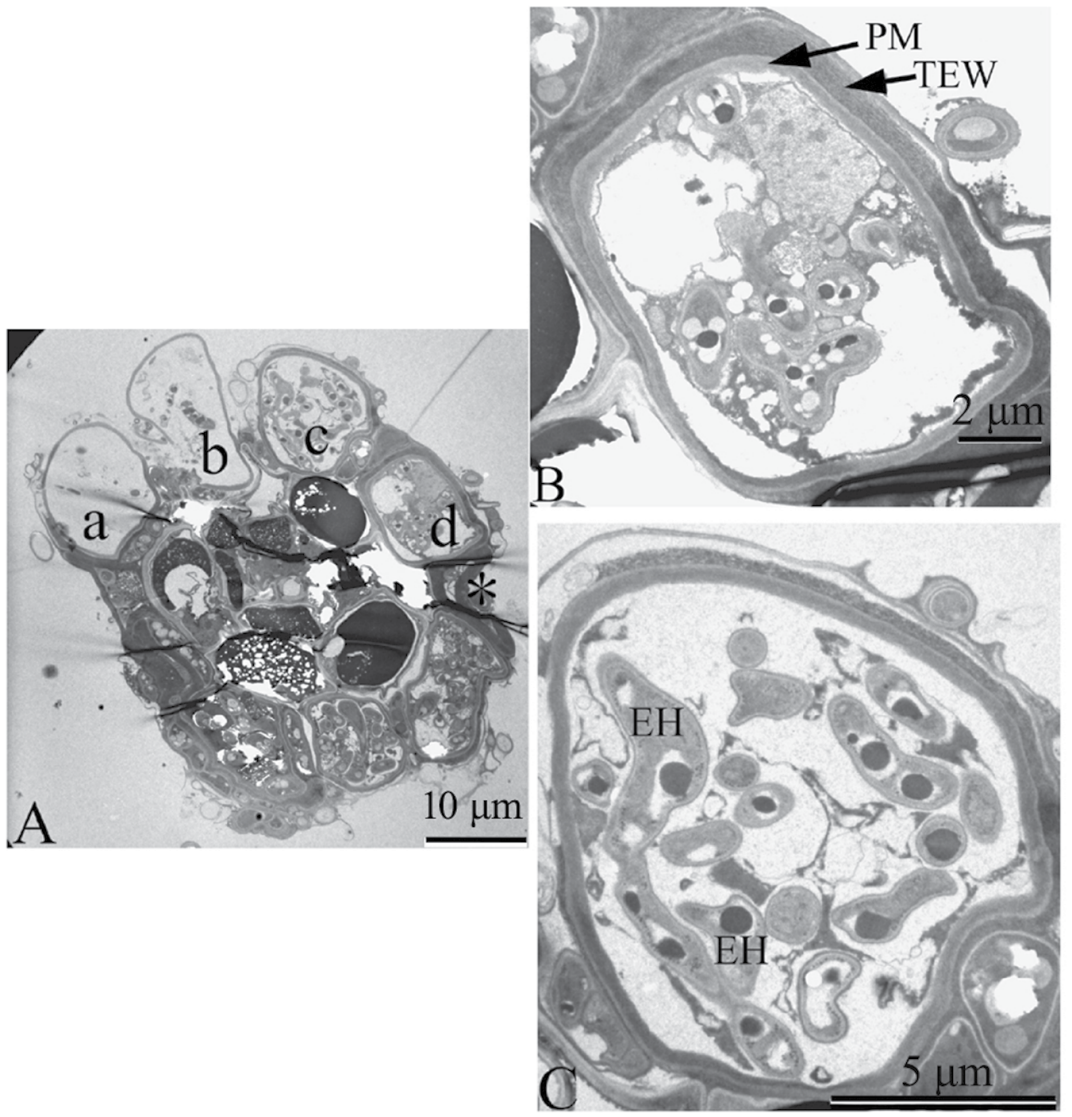
Observation of a section of ericoid mycorrhizal root from *V*. *uliginosum* by TEM. (**A**) Transverse sectional micrograph of an ericoid mycorrhizal root, indicating the epidermal cells fully colonized by the hyphae of *O*. *maius*. Two adjacent cells (b,c) in a transverse section had fungal complexes of different ages. (a,b) Cytoplasmic degeneration in the late stage of mycorrhizal colonization. (c) Mature colonization by hyphae that fill the cell. *A detached cell. (**B**,**C**) Higher magnified images of two cells (cell c,d in **A**). (**C**). PM: plasma membrane; TEW: thickened outer epidermal walls; EH: intracellular fungal hyphae.

TEM was used to observe the structure of hair roots of *V*. *uliginosum* and the ericoid mycorrhizae characteristics. The hair roots of *V*. *uliginosum* possess a small stele containing very small tracheids and sieve elements, and the vascular elements are enveloped by the cortex. The epidermis located in the outermost cell layer of the hair roots was colonized by EMF (Fig. 3). Observation by TEM showed that each epidermal cell represented a separate infection unit, and several adjacent epidermal cells possessed fungal complexes of different ages (Fig. 3A). In the mature stage of colonization, the plant cell was almost completely occupied by fungal hyphae with a discrete structural integrity within the plant cell. The hyphal complex was enveloped by a layer of the membrane structure and formed an independent hyphal coil occupying the cellular space (Fig. 3C). In some root cells colonized by ericoid mycorrhizae, we observed thickened outer epidermal walls (Fig. 3B). In the late stage of colonization, the plant and fungal tissues had deteriorated.

### Analysis of diversity of root-associated fungi via high-throughput sequencing

**Figure 4:**
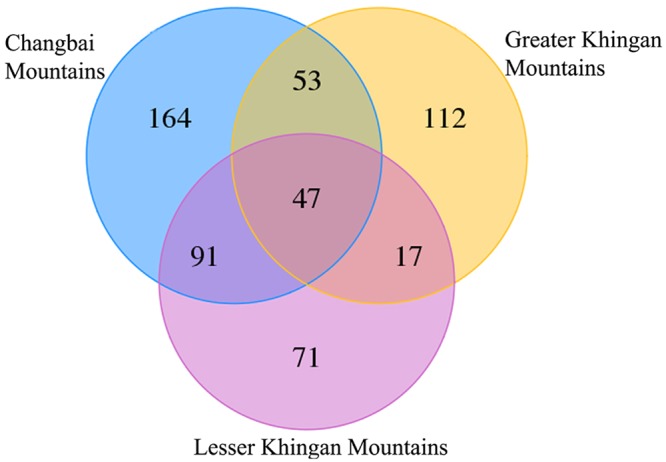
Venn diagram showing the specific and shared OTUs of different study areas.

Fungal communities in hair roots of *V*. *uliginosum* were analyzed using Illumina MiSeq sequencing. Successfully sequenced amplicons representing the total fungal community were acquired. We obtained 396,470 sequences of which 256,487 were retained after quality filtering. The average number of sequences per sample was 28,498 (ranging from 8,653 to 41,742). Using a 97% similarity cut-off, the sequences clustered into 555 OTUs. The mean number of OTUs per sample was 125. Venn diagrams showed the number of specific and shared OTUs by different study areas (Fig. 4). Results indicated that 47 OTUs were shared by three study areas (Fig. 4), which was 8.5% of total 555 OTUs. There were 164 OTUs specific to the Changbai Mountains, 112 specific to the Greater Khingan Mountains, and 71 specific to the Lesser Khingan Mountains, accounting for 29.5%, 20.2%, and 12.8% of total OTU richness, respectively. When 47 OTUs shared by all study areas were excluded, only 17 OTUs were found in both the Greater Khingan Mountains and the Lesser Khingan Mountains, which was far fewer than the number of shared OTUs between the Changbai Mountains and other study areas (the Greater Khingan Mountains and the Lesser Khingan Mountains). The number of both specific and total OTUs in the Changbai Mountains were much higher than in other study areas. The rarefaction curves of fungal OTUs in hair roots of *V*. *uliginosum* were acquired (Fig. S1), indicating that the OTU richness of the Changbai Mountains was higher than that of other study areas.

Among 555 OTUs, most belonged to Basidiomycota (27.4%) and Ascomycota (53.9%). Common classes were Agaricomycetes (22.5%), Archaeorhizomycetes (2.9%), Leotiomycetes (19.3%), Eurotiomycetes (4.3%), and Pezizomycetes (0.4%). Common fungal orders included Agaricales (6.7%), Archaeorhizomycetales (2.9%), Atheliales (2.9%), ectomycorrhizal fungi orders Russulales (2.0%), and putative EMF orders, such as Helotiales (16.6%), Hypocreales (3.8%), Sebacinales (3.1%), and Chaetothyriales (3.1%). Moreover, some fungal families, such as Herpotrichiellaceae (2.3%), Myxotrichaceae (0.5%), and Sebacinaceae (0.5%), which include EMF fungi, were also observed.

Of all 555 OTUs, 47.8% could be described to the genus level, making a total of 266 genera. Only 22 OTUs were assigned to putative EMF genera (Table 3). *Rhizoscyphus* and *Meliniomyces* dominated EMF communities with 45.5% of putative EMF OTUs, followed by *Clavaria* (18.2% of putative EMF OTUs), *Oidiodendron* and *Lachnum* (13.6% of putative EMF OTUs), *Acephala* and *Phialocephala* (4.5% of putative EMF OTUs). Among the putative EMF genera, the genera of *Acephala*, *Meliniomyces*, and *Phialocephala* belonged to DSE. The most common non-mycorrhizal OTUs were assigned to *Tylospora* (12.9% of all reads, 4 OTUs). Moreover, we also found putative ECM fungal genera, such as *Tomentella* (9166 sequences, 12 OTUs) and *Tomentellopsis* (17 reads, 1 OTU), and saprotrophic fungal genera, such as *Acremonium* (3 reads, 1 OTU) and *Myrothecium* (9 reads, 1 OTU).

**Table 3:** Putative EMF reads originated from different study areas.

OTU ID	Top hits in GenBank (Accession No.)	Similarity (%)	No. of reads		
			Changbai Mountains	Greater Khingan Mountains	Lesser Khingan Mountains
OTU25	*Oidiodendron maius* (HQ608115)	100	7	1403	1
OTU335	*Oidiodendron* sp. (AF062787)	96	3	1	0
OTU247	*Oidiodendron* sp. (AB986458)	96	4	11	0
OTU35	*Rhizoscyphus ericae* (AM084704)	96	0	737	0
OTU181	*Rhizoscyphus ericae* (AM887700)	99	16	0	8
OTU59	*Rhizoscyphu ericae* (KJ817285)	96	41	1	308
OTU430	*Rhizoscyphus monotropae* (KF359569)	99	0	3	0
OTU49	*Rhizoscyphus* sp. (JQ711893)	98	386	0	235
OTU141	*Lachnum* sp. (LC015693)	99	10	32	0
OTU237	*Lachnum* sp. (EU887662)	96	10	0	0
OTU53	*Lachnum virgineum* (KY322550)	99	300	90	4
OTU101	*Clavaria flavipe* (KC759451)	91	0	67	0
OTU530	*Clavaria flavipe* (KC759451)	92	0	8	0
OTU73	*Clavaria zollingeri* (AY854071)	98	0	179	0
OTU99	*Clavaria sphagnicola* (KC759455)	99	1	1	102
OTU450	*Acephala* sp. (FJ378718)	100	0	0	4
OTU28	*Phialocephala fortinii* (MF94865)	100	302	896	96
OTU82	*Meliniomyces* sp. (KC455342)	98	5	0	116
OTU116	*Meliniomyces* sp. (KC455343)	91	66	0	2
OTU150	*Meliniomyces variabilis* (NR_121313)	96	0	8	60
OTU203	*Meliniomyces* sp. (KC455343)	94	0	0	24
OTU261	*Meliniomyces_variabil* (KF156311)	100	8	0	15

Top hits in GenBank provided the best match of the representative sequences to GenBank, with accession numbers shown in brackets.

Of all 555 OTUs, 289 OUTs were assigned to putative life strategies using FunGuild. Saprotrophs were the most abundant, with 33.2% assigned OTUs, followed by symbiotrophs (24.2% assigned OTUs). Only 9.7% assigned OTUs were pathotrophs. In addition, 95 OTUs were assigned multiple trophic modes. Undefined saprotrophs (73 OTUs) were the largest guild in OTU richness, followed by ectomycorrhizal (54 OTUs), endophyte-soil saprotroph-undefined saprotroph (38 OTUs), and plant pathogen (19 OTUs). Only 3 OUTs of *Oidiodendron* genus were assigned to ericoid mycorrhizal guild. In addition, 6 OUTs of *Meliniomyces* genus and 3 OUTs of *Pezoloma* genus were assigned to the ectomycorrhizal-endophyte-ericoid mycorrhizal-litter saprotroph-orchid mycorrhizal guild.

*Acephala* was only found in the Lesser Khingan Mountains, and other putative EMF genera were distributed across all 3 study areas (Table 3). A large number of reads of genus *Rhizoscyphus* could be obtained from all 3 study areas. For *Oidiodendron*, most reads (99.4%) originated from the Greater Khingan Mountains. As a typical DSE, most reads (69.2%) of *P*. *fortinii* also came from the Greater Khingan Mountains (Table 3). Overall, there were more DSE reads from the Greater Khingan Mountains than from other study areas, as in the results describing the percent colonization of hair roots by DSE at different study areas.

**Figure 5:**
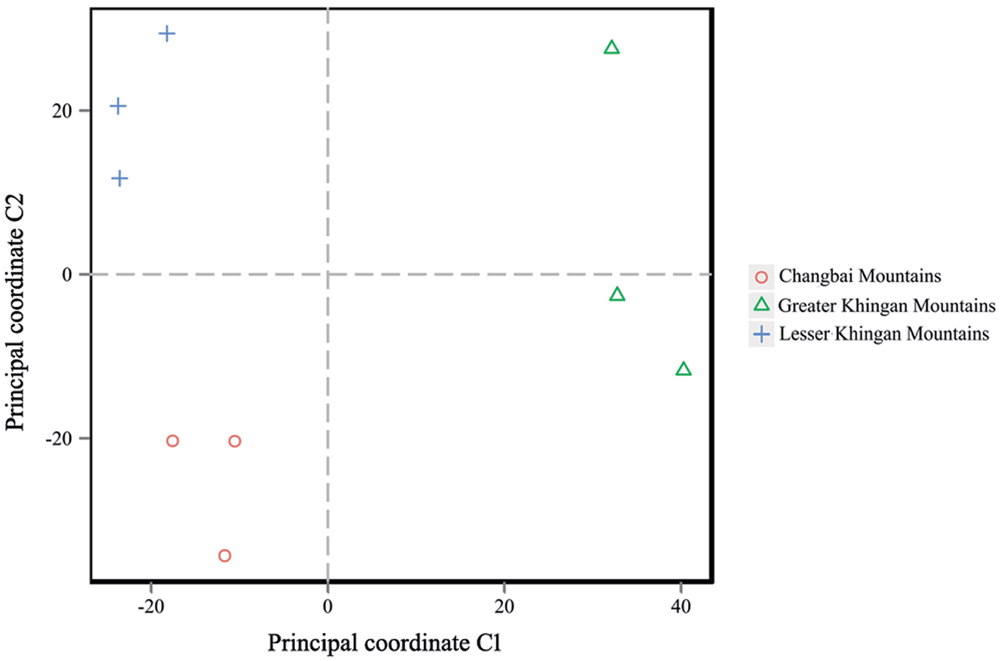
Principal coordinate analysis (PCoA) of fungal communities of *V*. *uliginosum* from different study areas.

**Figure 6:**
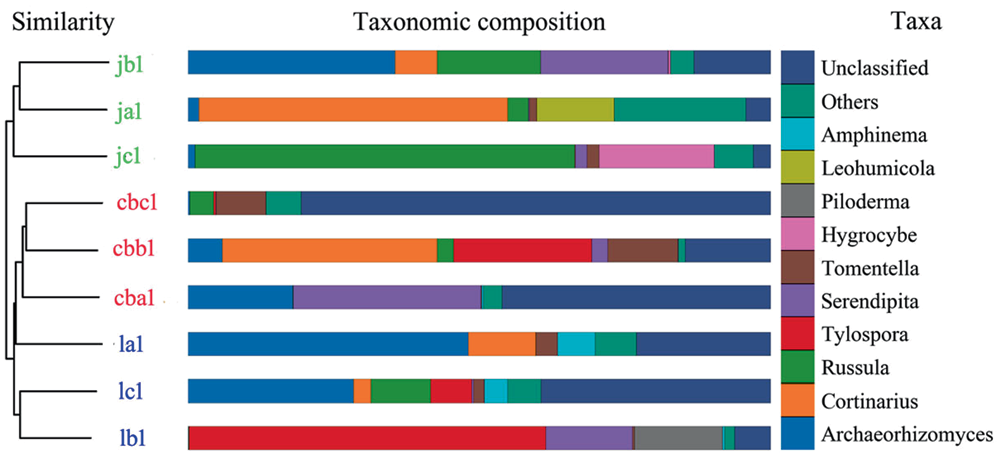
Taxonomic composition distribution of genus-level and UPGMA tree for different samples. Only the top 10 genera are shown for taxonomic composition distribution, and other genera were classified as “other”. In the UPGMA tree, samples originated from the Changbai Mountains, the Greater Khingan Mountains, and the Lesser Khingan Mountains, are shown as red, green, and blue, respectively.

Most of the samples from the same study area clustered together in the PCoA analyses (Fig. 5). However, one sample from the Greater Khingan Mountains was separated from the others, suggesting that this sample had a distinct fungal community. Similarly, most of samples from the same study area also grouped together in the UPGMA tree (Fig. 6). However, the la1 sample of the Lesser Khingan Mountains clustered together with those from the Changbai Mountains study area. In the taxonomic composition distribution figure of genus-level (Fig. 6), the abundances of genera *Archaeorhizomyces* and *Amphinema* of la1 sample were higher than those of other samples. We also evaluated Chao1 richness estimator, ACE richness estimator, and Shannon-Wiener diversity index of different study areas (Table S1) and no significant difference was found between different study areas under the Duncan’s multiple range test.

PERMANOVA analysis did not demonstrate significant difference (P > 0.05) between different study areas and latitude levels using both the Jaccard and Bray-Curtis distance matrix. There was a significant difference for fungal community structure between 2 pH levels (4.1–4.6 and 4.7–5.2) (P < 0.05) in PERMANOVA analysis when using the Jaccard matrix, but not when using the Bray-Curtis matrix.

## Discussion

### Isolation of root-associated fungi from *V*. *uliginosum*

*V*. *uliginosum* is an ecologically important heath species that is distributed across northern Asia, North America, and Europe. Although it is widely distributed in northeastern China^22^, its mycorrhizal status has rarely been investigated^3^. Here, we detected 16 fungal species from a small number of wild *V*. *uliginosum* samples collected from 12 study sites. These fungi included *O*. *maius*, a typical EMF, and several putative ericoid mycorrhizal fungal taxa, such as *Lachnum* sp., Sordariomycetes sp., *C*. *ericae*, and *P*. *fortinii*. *O*. *maius* and related taxa have been reported to form mycorrhizal associations with *Gaultheria shallon* in British Columbia, Canada^7^, with *V*. *corymbosum* in Quebec, Canada^24^, with *Calluna vulgaris* in Devon, UK^25^, with *C*. *vulgaris* in Italy^26^ and with *V*. *oldhamii* in Japan^27^. In China, *O*. *maius* has only been reported to form ericoid mycorrhizae with the genus *Rhododendron*^28–30^. As the second most common isolate, *Phomopsis* sp. did not form ericoid mycorrhizae in resynthesis trials. Several endophytes of the genus *Phomopsis* have been isolated from woody plants and orchidaceous plants, and these fungi are reported to assist the plant host in acquiring N^31,32^. *Phomopsis* sp. probably plays an important role in the hair roots of *V*. *uliginosum*. In addition, isolate 74 had an ITS sequence similarity of 97% with *U*. *isabellina* (Mucoromycotina). To our knowledge, no study reported the colonization of ericaceous plants by the endophytes or mycorrhizae of the genus *Umbelopsis*, and the majority of *Umbelopsis* colonizations appear to occur on orchids^33^. This is the first time, to our knowledge, that *U*. *isabellina* has been isolated from the hair roots of *V*. *uliginosum*. Very little is currently known regarding the function of the genus *Umbelopsis* in Orchidaceae and Ericaceae^33^. The colonization of *V*. *uliginosum* by *Umbelopsis* represents a unique type that requires further investigation.

Our study sites in the biodiverse Greater Khingan Mountains, Lesser Khingan Mountains, and Changbai Mountains^34,35^ encompassed almost all the main wild distributions of *V*. *uliginosum* in China^22^. While there is a strong possibility that the EMF diversity is also high in these regions on account of their geographical and environmental characteristics and high species diversity, the EMF isolated from the hair roots of *V*. *uliginosum* were not diverse. Only six species could be confirmed to have ericoid mycorrhizal status. In a previous study, the *R*. *ericae* aggregate, which contained closely related species in the order Helotiales, had a high proportion of isolated EMF^36^. However, the *R*. *ericae* aggregate was not isolated here. The strains of some genera, such as *Meliniomyces*, *Cadophora*, and *Geomyces*, have been reported as ericoid mycorrhizae and have been isolated from ericaceous plants roots^1,2,19,29^. However, none of these were isolated and neither were any basidiomycetes isolated in this study.

### Diversity of fungal OTUs of *V*. *uliginosum* based on high-throughput sequencing

In this study, diverse fungal taxa were found in hair roots of *V*. *uliginosum* via high-throughput sequencing. The order Helotiales, Hypocreales, Sebacinales, and Chaetothyriales, and the family Herpotrichiellaceae, Myxotrichaceae, and Sebacinaceae, which have been found in hair roots of many ericaceous plants^1,2,19,29^, were commonly associated with *V*. *uliginosum*. In addition, some putative EMF fungal genera, such as *Rhizoscyphus*, *Oidiodendron*, *Lachnum*, *Phialocephala*, *Clavaria*, *Acephala* and *Meliniomyces*, were observed. When excluding *Clavaria*, *Acephala*, and *Meliniomyces*, other genera were also isolated by MA and MMN media in the study.

Zhang *et al*. (2016) reported the diversity of root-associated fungi of *Vaccinium carlesii* and found fewer reads of *O*. *maius* and *R*. *ericae* via high-throughput sequencing. Rather, we found *R*. *ericae* and *O*. *maius* to be common species in high-throughput sequencing data (Table 3). We also obtained several isolates of *O*. *maius* using traditional isolation and culture methods. However, we did not determine the sequence of *Pleosporales* sp. from the high-throughput sequencing data. It is likely that we would obtain the sequences by testing more samples. In addition, we also found putative ECM fungal genera, ectomycorrhizal fungal order, and saprotrophic fungal genera, suggesting that there were diverse mycorrhizal fungi associated with *V*. *uliginosum* hair roots. These diverse mycorrhizal fungi may form complicated mycorrhizal networks and play an important role in the heathland ecosystem.

In PERMANOVA analysis, we obtained different results for the Jaccard and Bray-Curtis matrix. The Jaccard matrix analysis only accounts for the presence/absence of OTU, however the Bray-Curtis matrix analysis is more sensitive to changes in OTU abundance^37^. This suggested that the changes between 2 pH levels were more likely influenced by a loss or gain of species than by changes in abundance of present species.

In this study, we detected unclassified OUTs that represented approximately 12% of the total OTU richness. More EMF-related fungal taxa may be unearthed from the data concerning these unclassified OUTs. FUNGuild parsed a large portion of OTUs into ecologically meaningful categories. However, only few OTUs were assigned to ericoid mycorrhizal guild, suggesting that more EMF data need be supplemented in the FUNGuild database. Overall, high-throughput sequencing data provided a rough community-scale sketch of root-associated fungi of *V*. *uliginosum*, as previously reported in other plants^19,38^. We may establish more details in the rough community-scale sketch using more experiments, such as isolation-resynthesis and evaluation of growth promotion.

### Co-existence of EMF and DSE in the hair root of *V*. *uliginosum*

DSE have been found in more than 600 plant species and across 114 families of angiosperms and gymnosperms. They co-occur with other types of mycorrhizal fungi including AM fungi, EMF, ectomycorrhizal fungi, and orchid mycorrhizal fungi^39^. Read and Haselwandter^40^ discovered that AM fungi and DSE colonization were negatively correlated with altitude, suggesting that DSE are more prevalent than AM fungi at high elevations. In this study, the results of linear mixed model analysis on EMF and DSE colonization suggested that there were higher total colonization rates of DSE at high elevations. Scervino *et al*.^41^ found that the exudates of DSE could affect the development of AM fungi. DSE were also isolated from root tips colonized by ectomycorrhizal fungi^42^. In this study, both EMF and DSE were isolated from the hair roots of *V*. *uliginosum*, which is in agreement with Massicotte *et al*.^43^, who reported simultaneous ubiquitous EMF and DSE colonization in the roots of five ericaceous plants in Canada. Little is currently known regarding the mechanism influencing the balance between EMF and DSE. In commercial blueberry fields, the percentage of hair root cells with EMF was generally higher in organic than conventional fields, whereas colonization of DSE was higher in sandy rather than muck soils^44^. Based on the casual observations data during sample examination, we also found that EMF mainly colonized the mature roots of *V*. *uliginosum*, while colonization by DSE was more frequently observed in the old roots, suggesting that DSE could function in the recycling of nutrients from senescent roots back into the active roots, in addition to functioning in the uptake of nutrients and water^45^.

**Figure 7:**
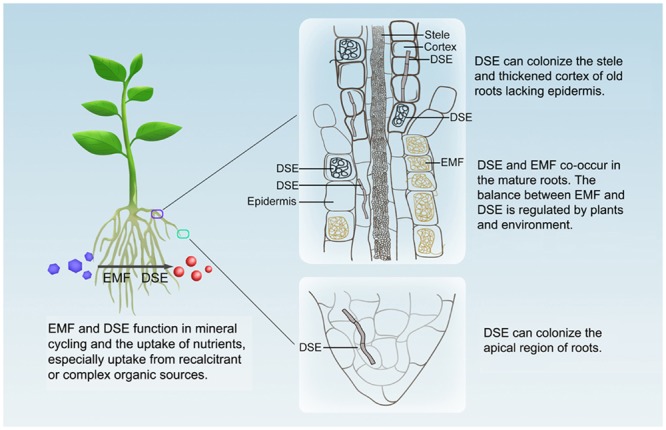
A putative model of the co-existence between EMF and DSE in the hair roots of *V*. *uliginosum*.

Based on the findings of previous reports^1,2,39,45^ and this study, we suggest a putative model for the co-existence of EMF and DSE in the hair roots of *V*. *uliginosum* (Fig. 7). In the apical region, a root cap possessing a discontinuous mucilage layer protects the root apical meristem, which comprises a small group of undifferentiated cells. DSE can colonize the apical region (Fig. 2E) whereas EMF cannot due to the lack of differentiated epidermal cells. In the mature roots, EMF are generally dominant and colonize the epidermal cells, and a small number of DSE can also colonize and invade the different tissues of the hair roots. The balance between EMF and DSE is regulated by plants and the environment. In the old roots, the epidermal layer disappears, and thickened cortical cells then become the outer surfaces of the roots. DSE can colonize the different root tissues. The aging of the roots may favor DSE colonization.

Although EMF and DSE can co-exist in the same hair roots, their interactions with the plant roots differ. EMF occur in the epidermal cells, whereas DSE colonize the root tissues intracellularly and intercellularly; thus, DSE not only interface via the epidermal cells, but also grow into the sieve elements, which are specific tissues for carbon transport^46^. The result is that the DSE are able to exert their biological functions more advantageously. However, a shift from mutualism to parasitism for DSE will probably occur if the excessive hyphae of DSE consume large amounts of nutrients.

A wide range of active enzymes, including cellulose, laccase, pectinase, xylanase, proteinase, and polyphenol oxidase, have been reported for both DSE and EMF^1,39^ and confer the potential ability to degrade complex organic material. Moreover, EMF can produce chitinase, which is likely to be unfavorable for the co-existence of DSE and EMF in the same or adjacent cells of the hair roots^47^. DSE occasionally have a negative effect on nutrient uptake when co-occurring with other mycorrhizal types. A strain of *P*. *fortinii* (DSE) suppressed the positive effect of *O*. *maius* (EMF) on P and N uptake when they were co-inoculated in *Rhododendron* plants^48^. Additionally, DSE in the mantles of inoculated seedlings forming ectomycorrhizal symbioses with *Amphinema byssoides* were associated with a reduction in the uptake of ^15^N^49^.

Ericoid mycorrhizae possibly constitute the youngest main mycorrhizal group, originating from the most recent common ancestor of the Ericaceae^2^. Ericaceous plants can exist in conditions of extreme mineral N and P limitation owing to this plant/fungal symbiosis^50^. DSE may exist with a versatile manner along the mutualism-parasitism continuum, and thus this symbiosis may be considered as mycorrhiza under some conditions^51^; however, whether the functions of DSE complement those of the mycorrhizal fungi remains unknown. In general, DSE possibly function as a back-up system when EMF colonization cannot occur in specific environmental conditions^52^. We propose that the co-existence of EMF and DSE is an optimal choice in the evolutionary process for ericaceous plants under environmental stress. Overall, the co-existence of EMF and DSE in the hair roots of *V*. *uliginosum* might be ecologically significant. The number of samples is small in this study, and a larger number of environmental factors may influence on the colonization and co-existence of EMF and DSE in hair root of *V*. *uliginosum*. Many questions still remain regarding the association between EMF and DSE; and a further detailed examination of the interactions and ecological functions of EMF and DSE is required.

## Materials and Methods

### Study area and sampling

This study was conducted in 3 study areas, including the Greater Khingan Mountains (ja–jf study sites), the Lesser Khingan Mountains (la, lb, and lc study sites) in Heilongjiang province, and in the Changbai Mountains (cba, cbb, and cbc study sites) in Jilin Province, northeastern China. The location and other environmental characteristics of the study sites are presented in Table 4.

**Table 4:** Characteristics of the study sites in northeastern China.

Study site	Changbai Mountains			Greater Khingan Mountains						Lesser Khingan Mountains		
	cba	cbb	cbc	ja	jb	jc	jd	je	jf	la	lb	lc
Location	128°15′38″E; 42°03′36″N	128°16′23″E; 42°05′00″N	128°13′04″E; 42°06′42″N	124°07′00″E; 50°17′22″N	124°06′48″E; 50°17′03″N	124°07′05″E; 50°17′30″N	124°07′02″E; 50°17′21″N	124°06′01″E; 50°16′22″N	124°06′27″E; 50°20′32″N	129°16′42″E; 48°11′17″N	129°13′47″E; 48°12′36″N	129°13′39″E; 48°09′55″N
Climate	temperate continental monsoon climate			cold continental monsoon climate						continental monsoon climate		
Frost-free days	100			85–130						106		
Mean annual rainfall	700–1400 mm			400–800 mm						550–600 mm		
Mean annual temperature	3–7 °C			−2.8 °C						0–2 °C		
pH	4.9	4.7	4.7	5.5	4.9	5.7	—	—	—	4.4	4.5	4.5
Water-soluble salt (μs/cm)	66	224	182	186	93	171	—	—	—	190	294	300
CaCO_3_ (g/kg)	6.7	23.3	27.9	37.7	26.6	26.3	—	—	—	30.2	55.0	23.8
Organic matter (g/kg)	78	615	661	394	174	294	—	—	—	606	704	575
Total N (g/kg)	1.30	1.40	1.39	1.53	1.47	1.33	—	—	—	1.27	1.30	1.30
Total P (g/kg)	0.18	1.21	0.88	2.28	1.31	2.24	—	—	—	1.09	1.22	1.15
Total K (g/kg)	37.20	5.82	6.14	8.32	19.60	11.10	—	—	—	5.67	8.97	6.21

^—^The values for the samples were not tested.

*V*. *uliginosum* is widely distributed in open fields or in the understories of forests where it grows in strips or masses. The samples of jd, je, and jf study sites were collected on September, 2013, and other samples collection occurred during August of 2014. To minimize damage to the roots, whole plants were removed with intact soil cores, placed into containers, and transported back to the laboratory. Ten root samples were collected from each study site, finally, 120 samples were obtained. Water-soluble salt, pH values, calcium carbonate (CaCO_3_), organic matter, and total nitrogen (N), phosphorous (P), and potassium (K) in the soil were measured according to Ajmal and Khan^21^ and the results are presented in Table 4.

### Isolation and observation of endophytic fungal colonization

Fungi were isolated from sterilized root pieces using the direct plating method of Pearson and Read (1973). Sterilized root pieces (3–5 mm long) were placed in 9.0 cm diameter Petri dishes containing Melin-Norkrans (MMN) medium or Martin’s rose-bengal agar (MA) medium (ratio of 1:1)^53^. Petri dishes were incubated in the dark at 25 °C for two weeks and observed daily for hyphal emergence. Fast-growing fungi from the root pieces were discarded, whereas slow-growing fungal colonies were sub-cultured onto malt extract agar (MEA) medium. Mycelia were transferred to new plates containing the same medium and sub-cultured 3–4 times in order to obtain a pure isolate. The cultures were maintained by sub-culturing on MEA medium.

The morphological characters of the colonies were observed on potato dextrose agar (PDA) plates and characterized according to the method of Photita *et al*.^54^. The growth rates of the fungal colonies were recorded after one week along two diametrical lines. Barnett and Hunter^55^ were referenced for the morphological identification. The endophytic fungi were preliminarily divided into different groups based on the culture characteristics, including colony shape, height and color of the aerial hyphae, base color, growth rate, margin, surface texture, and depth of growth into the medium.

Root samples for mycorrhizal observation were stained as described previously by Phillips and Hayman^56^ with minor modifications. Briefly, the clean roots were cleared with 10% potassium hydroxide (w/v) for two days, acidified with 1% HCl (v/v) for one day, and stained with 0.05% trypan blue lactic acid solution (w/v) for five days. The excess stain was removed in clear lactophenol. The stained roots were then observed under a light microscope (TE2000, Nikon, Tokyo, Japan) and images were recorded using NIS-Elements software (Nikon, Tokyo, Japan).

### Fungal colonization evaluation

Visual examination of colonization was executed following the magnified intersections method^57^, using 400× magnification. Ninety samples of 9 study sites (cba, cbb, cbc, ja, jb, jc, la, lb, and lc) (10 samples per study site) were tested, and 80 to 100 intersections per root segment were scored depending on the root segment size (480–600 intersections per hair root sample). Young, mature, and old roots were determined by color (from white or light yellow to yellowish-brown), diameter (from small to large), lignifying level (from low to high), and hardness (from soft to hard). The percentage of root length colonized by EMF^58^ and the percentage of DSE total colonization^59^ were calculated. Both dark septate hyphae and microsclerotia were calculated for the percentage of DSE total colonization.

Pearson’s correlation was used to assess the relationship between EMF and DSE variables via SPSS Version 22.0 (IBM Corp., Armonk, NY, USA). Linear mixed model analysis was used to evaluate the impact of several environmental factors (pH, latitude, EMF colonization rates or total colonization rates of DSE) on EMF or DSE colonization rates using SPSS Version 22.0. Study site was used as a random factor, pH, latitude, total colonization rates of DSE or EMF colonization rates were used as fixed factors.

### Ribosomal DNA amplification and sequencing analysis

Sequence-based methods were used to assist in fungal identification. The 280 slow-growing fungi isolated from the roots of *V*. *uliginosum* were divided into 13 groups on the basis of macroscopic morphology and microscopic examination. Fifty-seven strains were selected from the 13 groups for culture in liquid MEA for 1–2 weeks at 28 °C, and pure strains were selected for total nucleic acid extraction following the modified method of Yang *et al*.^60^.

The ribosomal internal transcribed spacer (ITS) region was amplified with the primer pair ITS1/ITS4 as described by White *et al*.^61^. PCR was performed as follows: the reaction mixture had a total volume of 50 μL, which contained 5 μL 10× buffer (with Mg^2+^), 3.2 μL (10 μM) dNTPs (TaKaRa, Kusatsu, Shiga, Japan), 1 μL (5 μM) each primer (Sangon, Shanghai, China), 5 U LA *Taq* DNA polymerase (TaKaRa, Kusatsu, Shiga, Japan), 37.4 μL H_2_O, and 2 μL genomic DNA. The samples were incubated in a thermal cycler at 94 °C for 3 min, followed by 30 cycles of 94 °C for 30 s, 54 °C for 30 s, 72 °C for 1 min; and a final extension step at 72 °C for 7 min. The PCR products were examined by electrophoresis in 1.2% agarose gel and stained with GelRed (Biotium, Fremont, CA, USA). The expected bands were purified using a gel extraction kit (Sangon, Shanghai, China). Sequencing was performed using an ABI 3730 automated sequencer (Applied Biosystems, Foster City, CA, USA).

The nucleotide acid sequences were compared with those in GenBank and the UNITE database using the BLAST facility^62,63^. The sequences were submitted to GenBank with the accession numbers KY910182-KY910238.

### Resynthesis of mycorrhizae

Fungal isolates were assessed for their ability to form ericoid mycorrhizae by inoculating *V*. *uliginosum* seedlings. Micropropagated plantlets of *V*. *uliginosum* were used as the test plants. Sterile seedlings were transplanted into culture bottles containing the sterilized soil mixture (peat moss/vermiculite [1:1, v/v], autoclaved twice for 40 min) and woody plant medium (Yutong, Shanghai, China). Inoculations were performed following the method described in Vohník *et al*.^17^ with some modifications. A 4 mm^2^ plug of actively growing mycelium was placed 2 mm below each seedling; uninoculated controls received a 4 mm^2^ plug of sterile MMN agar. The culture bottles were covered with a sterile filtration membrane to avoid contamination. The seedlings were maintained in a growth chamber under a 22 °C, 16 h/8 h day/night cycle and irradiation of 70 µmol m^−2^ s^−1^.

The roots were stained with trypan blue one and four months later after inoculation. The colonization status of the root cells was observed under a light microscope. Additionally, the ultrastructural features of the inoculated and non-inoculated root samples of *V*. *uliginosum* were investigated by electron microscopy after fixation and embedding of the specimens in Araldite^64^.

### High-throughput sequencing and bioinformatic analysis

The samples (cba1, cbb1, cbc1, ja1, jb1, jc1, la1, lb1, and lc1) were also analyzed by high-throughput sequencing. The samples were carefully washed to remove all soil and plant debris. The samples were put into 50 mL centrifuge tube and preserved at −80 °C.

Total nucleic acid was extracted using CTAB methods with slight modifications^19^. Briefly, hair root tissues were put into a sterile centrifuge tube containing CTAB buffer and ground with a plastic pestle on ice, incubate the sample for 15 min at 65 °C. Extract twice with chloroform/isoamyl alcohol (24:1), separating the phases by spinning, add ethanol to the water phase and mix well, precipitate nucleic acid by spinning. Wash the pellet with 70% ethanol, and dissolve nucleic acid in 30 µl of ddH_2_O. PCR amplification were described in detail by Zhang *et al*. (2016). Briefly, the ITS1F-ITS2 region of fungi were amplified by PCR with an annealing temperature 55 °C. PCR were performed using FastPfu Polymerase (TransGen Biotech, Beijing, China). Purified amplicons were pooled in equi-molar and paired-end sequenced using the strategies of PE250 (paired-end sequenced 250 × 2) on an Illumina MiSeq platform according to the standard protocols.

After paired-end reads were generated, the reads with sequencing adapters (5 bases overlapped by reads and adapter with maximal 3 bases mismatch allowed), ambiguous base, and low complexity (reads with 10 consecutive same bases) were filtered out. According to the relationships between the paired-end reads and overlapping region, pairs of reads were merged using FLASH 1.2.3^65^. During the merging process, the minimal overlapping length was 15 bp and the mismatching ratio of overlapped region was no more than 0.1. Pre-filtered reads were dereplicated and singletons were removed using USEARCH 7.0^66^. These reads were clustered into OTUs at a 97% similarity cutoff following the UPARSE pipeline^66^. Chimeras were filtered out using UCHIME (v4.2.40)^67^. The phylogenetic affiliation of each sequence was analyzed by RDP Classifier (http://rdp.cme.msu.edu/) using confidence threshold of 80%. OTU table was obtained using QIIME 1.7^68^. The OTUs were assigned to saprotrophic, symbiotrophic, and pathotrophic trophic modes using FUNGuild^69^. The rarefaction curves of fungal OTUs was generated by R (version 3.2)^70^. The Venn diagram was constructed by VennDiagram package (version 1.6.20)^71^.

The Chao1 richness estimator, ACE richness estimator, and Shannon-Wiener diversity index were generated by Mothur 1.3^72^. The Bray–Curtis dissimilarity index was used to generate community distance matrices. Based on the relative abundances of the OTUs per sample and Bray-Curtis distances, a principal co-ordinate analysis (PCoA) was conducted using QIIME 1.7^68^. Unweighted pair group method with arithmetic mean (UPGMA) cluster analysis was performed based on distance coefficient. Taxonomic composition distribution of genus-level for different samples was generated by R (version 3.2). Only the top 10 genera are shown, and other genera were classified as “other.” The significance of differences in fungal community structure among 3 study areas, 3 pH levels (4.1–4.6, 4.7–5.2, 5.3–5.8) and 3 latitude levels (40.1–45.0, 45.1–50.0, 50.1–55.0) were evaluated using PERMANOVA analysis^73^ based on the Jaccard and Bray-Curtis distance matrix by PAST 3.20^74^.

## Electronic supplementary material

Supplementary information for Table S1 and Figure S1
