# Author Correction: Diversity and characteristics of colonization of root-associated fungi of *Vaccinium uliginosum*

**DOI:** 10.1038/s41598-020-66544-2

**Published:** 2020-06-10

**Authors:** Hongyi Yang, Xingyu Zhao, Changli Liu, Long Bai, Min Zhao, Lili Li

**Affiliations:** 1 College of Life Sciences, Northeast Forestry University, Harbin, 150040 China; 2 Institute of Forestry Science of Heilongjiang Province, Harbin, 150081 China

Correction to: *Scientific Reports* 10.1038/s41598-018-33634-1, published online 16 October 2018

Data Availability statement was omitted in this Article. It is included below:

## Data Availability

Raw sequencing data generated in this study are available in the Short Read Archive under accession PRJNA633221.

