# Supplementary material for: Diversity and characteristics of colonization of root-associated fungi of Vaccinium uliginosum
Source: Sci Rep. 2018 Oct 16;8:15283. doi: 10.1038/s41598-018-33634-1 (PMC6191440; doi:10.1038/s41598-018-33634-1)
Supplement: Supplementary file 1 — Supplementary information for Table S1 and Figure S1 [file 41598_2018_33634_MOESM1_ESM.doc]

Title: Diversity and characteristics of colonization of root-associated fungi of *Vaccinium uliginosum*

Authors: Hongyi Yang^1^, Xingyu Zhao^1^, Changli Liu^1^, Long Bai^1^, Min Zhao^1*^, Lili Li^2*^

Table S1 Diversity and richness index of fungal community from 3 study sites. The numbers represent the mean values ± SD.

| Study areas | OTUs | ACE | Chao1 | Shannon-Wiener |
| --- | --- | --- | --- | --- |
| Changbai Mountains | 173±71 | 197.0± 62.8 | 193.5± 62.3 | 2.06±0.21 |
| Greater Khingan Mountains | 102±32 | 113.2± 33.0 | 110.7± 32.1 | 1.75±0.31 |
| Lesser Khingan Mountains | 101±22 | 117.6± 25.6 | 116.7± 28.2 | 2.06±0.61 |


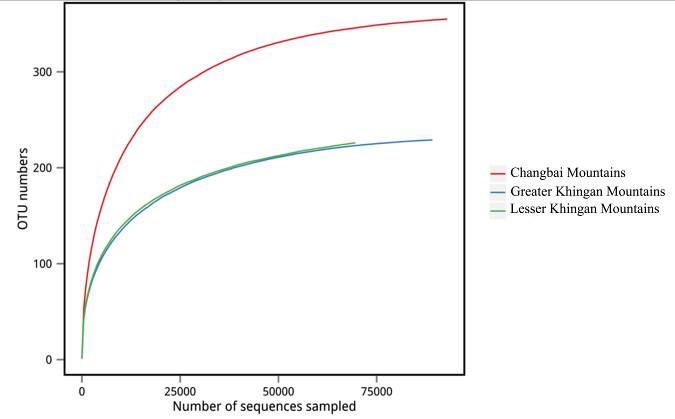


Figure S1 Rarefaction curves of fungal OTUs from hair roots of *V. uliginosum* at different study areas. Three study sites are shown as different colors.
